# A Bibliometric Analysis of Nurses' Job Satisfaction From 2004 to 2023

**DOI:** 10.1155/jonm/4285361

**Published:** 2025-04-30

**Authors:** Tianli Huang, Yan Wu

**Affiliations:** Department of Nursing, Zhongshan Hospital, Fudan University, Shanghai 200032, China

**Keywords:** bibliometric analysis, job satisfaction, nurses, Web of Science

## Abstract

**Aim:** To conduct a bibliometric analysis of the nurses' job satisfaction from 2004 to 2023.

**Design:** The bibliometric and visual analysis was performed in January 2024.

**Methods:** Bibliometric approaches were applied to analyse 11,993 articles, utilising R and VOSviewer software.

**Results:** Articles published by 24,155 authors from 1735 distinct sources between 2004 and 2023 were retrieved from the Web of Science and incorporated into the research's purview. The most productive nation and institution correspondingly were the United States and the University of Toronto. The leading scholars in this sphere were Spence Laschinger, Heather K, Labrague, Leodoro J, and Rodwell, John according to Price's Law, author co-citation and bibliographic-coupling network analysis. 14,152 keywords about nurses' job satisfaction study were discovered in this research. The most common keywords encompassed “job satisfaction,” “nurses,” “burnout,” “turnover,” and “intention” It was also observed that while trend topics like “work engagement” “COVID-19” and “grit” have gained popularity recently, the most commonly employed trend topics in earlier years included “empirical research report” “longitudinal study,” and “organizational characteristics.”

**Conclusion:** Research on nurses' job satisfaction remains relatively limited and requires more attention, especially in developing countries. Developed countries, especially the United Kingdom and the United States, are the main contributors to nurse job satisfaction research. In the early days, nurse job satisfaction research mainly focused on the current status and influencing factors of nurse job satisfaction in different medical organizations, nurse groups or departments, while more researchers have recently paid more attention to research on specific issues emerging in this field, such as the impact of COVID-19 on nurse job satisfaction and turnover. In addition, scholars in the field of nurse job satisfaction focus on finding the real determinants of job satisfaction of adult practicing nurses, such as interpersonal value consistency, human resource management, and the impact of job satisfaction of adult nurses in different medical environments. Topics such as “perseverance,” “COVID-19” and “work engagement” may be potential focuses for future research. Furthermore, transnational research should be given greater emphasis to investigate whether the major factors and effective interferences of nurses' job satisfaction differ between cultures and more multicenter as well as big sample studies should be conducted to efficiently improve nurses' job satisfaction.

**Impact:** This study used bibliometric analysis to examine the most contributing nations, institutions, authors, trend topics, and research focus. Data on the present state of nurses' job satisfaction research, including its knowledge maps, study emphasis, and thematic trends are few. The findings of this research can lay a strong basis for future research and offer direction.

**No Patient or Public Contribution:** There were no humankind subjects in the bibliometric analysis of published papers.

## 1. Introduction

Job satisfaction is described as an emotional, subjective, and multidimensional state that results from job evaluation and multiple elements and ranges from discontent to satisfaction [1–4]. In the past, many researchers have studied and defined the concept of job satisfaction. Based on Maslow's hierarchy of needs, job satisfaction is defined as the alignment between an individual's needs and the perceived ability of a career to fulfill those needs [5]. Wolf similarly characterizes job satisfaction as the fulfillment of needs [6]. The two-factor theory emphasizes employee motivation in the context of job satisfaction. Gruneberg [7] describes job satisfaction as the overall feelings employees have toward their work. Smith et al. [8] further define it as employees' emotional reactions to various aspects of their jobs. Butler and Banik [9] refer to job satisfaction as the satisfaction and self-fulfillment derived from the work environment [10].

From a motivational theory perspective, job satisfaction can be understood as the degree to which personal expectations are met in one's current role [11]. Locke [12] originally defined job satisfaction as “a pleasant emotional state resulting from the evaluation of one's job in relation to personal work values,” later refining this definition to encompass the positive feelings resulting from job evaluations [13]. In the specific context of nursing, Atwood and Hinshaw [14] describe nurse job satisfaction as the subjective perception of one's job and working conditions, while Spector [15] characterizes it as an emotional response to work and the environment. Mueller and McCloskey [16] define nurse job satisfaction as the degree of positive emotional orientation toward one's employment [17].

Nurse job satisfaction is critical in healthcare management and delivery worldwide. It significantly influences the quality of medical care; satisfied employees are more productive, creative, and likely to remain with their organizations [18]. Moreover, job satisfaction is a global concern due to its potential impact on patient care quality and safety [19, 20]. Low job satisfaction among nurses can adversely affect their performance and the quality of care they provide [21]. Thus, implementing organizational strategies to prevent burnout and enhance job satisfaction can lead to improved patient care [22]. Additionally, nursing job satisfaction has been linked to patient satisfaction [18, 23].

Furthermore, nurse job satisfaction is crucial for nurse retention. In recent years, many nurses have left their positions due to challenging working conditions [24, 25] and unsatisfactory workplace environments [26, 27], contributing to a growing nursing shortage. High job satisfaction correlates with efficient performance and a greater likelihood of continued employment [28]. Moreover, low job satisfaction is not only related to employee turnover and turnover intention [29], but also to employee mental health and burnout [30]. Because nurses make up the majority of healthcare workers in nearly every nation and new nurses' level of job satisfaction is taken as the most significant element affecting their retention [31]. In addition, it is because the need for nurses has been growing with an ageing population, pandemics, and high attrition rates, making them indispensable in the provision of care in hospitals, nursing institutions, and the community [32, 33]. Understanding the research hotspots and emerging trends of nurses' job satisfaction is imperative for healthcare administrators [34], policymakers, and researchers to devise strategies that enhance workplace environments and improve patient outcomes [35].

In the complex and messy existing research in this field, traditional critical research can no longer meet the requirements of sorting out more systematic and comprehensive knowledge information. Traditional research mainly relies on experts in the subject to conduct qualitative research based on their respective understanding of the development of the subject, but it is inevitable that there will be omissions and subjective biases. For new forces in the subject field, due to their lack of professional quality, it is a very difficult task to accurately identify key documents in a large number of documents in this field. The method of bibliometrics provides a new idea for this type of research. This method can effectively avoid the emergence of the above two problems and reflect the history of the subject more objectively and truthfully [36]. A strong statistical analysis tool, bibliometric analysis helps to perform an objective, scientific quantitative analysis of the articles [37, 38]. This allows us to thoroughly and methodically understand a research domain, as well as hotspots and emerging trends. With statistical techniques, bibliometric analysis involves charting out research trends, producing a profile of scientific research on a particular topic, and detecting qualitative as well as quantitative shifts in a scientific study [39]. Performance analysis and scientific mapping are the two fundamental bibliometric analysis procedures. The number of studies and citations is essentially the focus of performance analysis, which generates specific bibliometric indicators and gives researchers a comprehensive examination. Based on the goal of the study, researchers can opt to analyse each of these structures simultaneously or concentrate on a few. This study used VOSviewer and Bibliometrix in R language for bibliometric analysis tools. VOSviewe, developed in 2010, is written in JAVA and has better processing and visualization effects on large amounts of data [40]. Bibliometrix is an open-source R package designed for comprehensive scientific mapping analysis. It offers a structured workflow for conducting bibliometric analysis, benefiting from R's flexibility and ease of upgrades. This integration allows for compatibility with other statistical packages, enhancing its analytical capabilities [41].

The present status and the development of nurses' job satisfaction research have been little analysed in prior studies. By employing bibliometric analysis, the research elaborates the focus and thematic trends in nurses' job satisfaction studies, filling the vacuum in the literature and providing an interpretation of the present data. This study analyses conceptual structures and dynamics and the study's findings offer an updated perspective on the trend topics in nurses' job satisfaction and inspire researchers' creative ideas for future research. Additionally, this research can help professionals and academics identify significant influences from authors, journals, countries, institutions, and research topics [42].

## 2. The Study

### 2.1. Design

In the study, a descriptive bibliometric analysis of the publications on nurses' job satisfaction was carried out in January 2024. Researchers could discover the publication trends related to a certain issue and gain a more comprehensive understanding of the study field by utilising the bibliometric analysis approach [43]. In order to have in-depth knowledge of nurses' job satisfaction between January 1, 2004 and December 31, 2023, it was utilised in this study [44].

#### 2.1.1. Research Questions

• How many publications and citations occur annually?
• What is the keywords' co-occurrence network for nurses' job satisfaction?
• What is the keyword thematic map for nurses' job satisfaction research?
• Which terms are trending in research on nurses' job satisfaction?

### 2.2. Sample/Participants

The research was conducted by analysing 605 articles about nurses' job satisfaction from the Web of Science database [45].

### 2.3. Data Collection

For the sake of guaranteeing the scope and reliability of the information, all bibliometric data in the text format was attained from the Web of Science database(core collection), and SSCI as well as SCI-Expended were chosen as the indices. In addition to WosCC, we also use Scopus and PubMed databases to ensure the comprehensiveness of research literature data. After determining the search database. First, according to the research topic, we determine the search terms such as “nurse” and “job satisfaction” for the subsequent construction of corresponding search formulas in the literature database. Secondly, in WosCC, we merge the search results of the three search methods of subject search, title search and abstract search to finally form a search formula to ensure the comprehensiveness of the search results. The construction process of the search formula in WosCC is detailed in Table S1 in the supporting information. After the preliminary search based on the clue type, the articles that are not related to the research are eliminated according to the literature category classification in the WosCC database and the abstracts of the search result articles are read. Literature that is not related to the research, such as articles in the fields of robotics and forestry, a total of 443 articles, are excluded. Finally, a total of 5634 articles are retrieved and used for research in WosCC [46]. In the Scopus database, a search formula was constructed using title-abstract search. The construction process of the search formula is detailed in Table S2 in the supporting information. A total of 4263 articles were retrieved. According to the literature keyword classification in the Scopus database, articles with classifications not related to the research were eliminated and the titles and abstracts of the retrieved articles were read. After excluding 387 articles not related to the research, a total of 3876 articles were finally retrieved and used for research in the Scopus database. In the PubMed database, a search formula was constructed using MeSH subject search and title/abstract search. The final search process is detailed in Table S3 in the supporting information. After excluding 278 irrelevant articles, a total of 4160 articles were finally retrieved and used for research in the PubMed database [47].

**Figure 1:**
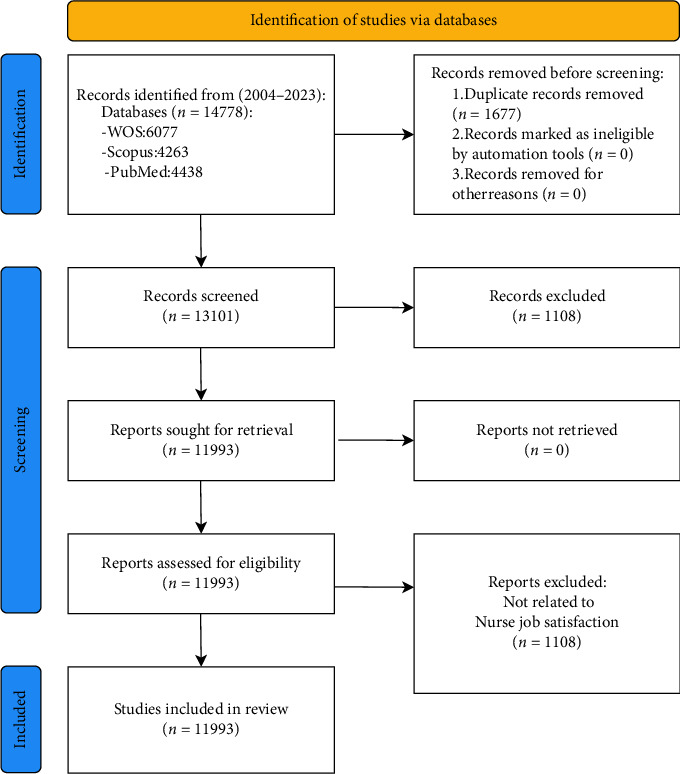
PRISMA search flowchart.

Finally, the literature retrieved from the three databases was merged and 1677 duplicate searched articles in the three databases were removed. Finally, a total of 11,993 literature data were included for research. The literature search process is detailed in Figure 1.

### 2.4. Ethical Considerations

The research was not needed for approval from the Research Ethical Committee because there were no humankind subjects in the bibliometric analysis of published papers [48].

### 2.5. Data Analysis

Every piece of data associated with the document settings, consisting of the abstract, keywords, bibliographic information, citation information, entire record, and cited references, was exported. HistCite as well as Excel were employed to accomplish the data and visual analysis of the publishing time, nations, yearly volume of publications, periodicals, organizations, authors, language, and citations. Bibliometrix Package in R was utilized to shape the global map displaying publication distribution as well as nation co-authorship, core sources by Bradford's Law, the patterns of journals' yearly total publications, the knowledge map of the institutions' cooperation network, visualized word clouds of keywords, thematic maps, and trend topics. Additionally, VOSviewer software was applied to illustrate the co-occurrence map of keywords based on the titles as well as abstracts, the co-citation and bibliographic-coupling maps of authors [49].

### 2.6. Validity and Reliability/Rigour

The selection procedure for these studies is repeatable and every citation was exported in TXT format from the Web of Science database. Scopus database data was exported from the database in CSV format. Pubmed database data was exported from the database in nbin format. CiteSpace was used to merge and remove duplicate literature data from the three databases. A third party was invited to participate in arbitration after two researchers separately included and removed the publications based on any discrepancies. Furthermore, the dependability of the results was ensured by the use of quantitative data for all analyses [50].

## 3. Results

### 3.1. Descriptive Analysis

According to the selection criteria, a total of 11,993 papers on nurse job satisfaction were included in the three databases from 2004 to 2023, written by 24,155 authors from 1001 institutions in 89 countries, published in 1735 journals, and cited 240,194 articles. Most of the published articles (94.1%) were written in English, and the remaining approximately 5.9% were written in Korean, German, Spanish, Italian, Portuguese, Turkish, French, Japanese, and Polish [51].

#### 3.1.1. Articles Distribution Throughout Time as well as Between Nations

**Figure 2:**
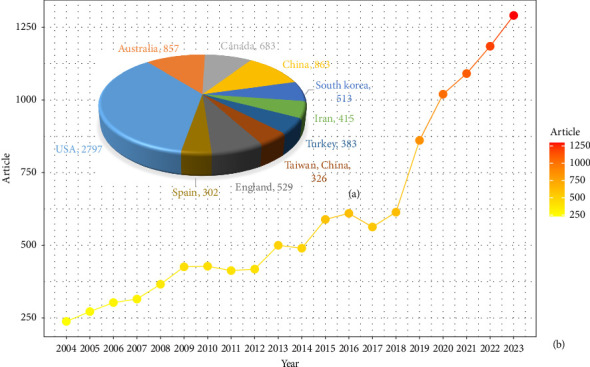
(a) Growth trend of the WoS articles on nurses' job satisfaction research. (b) The top 10 prolific countries based on the number of publications related to nurses' job satisfaction.

Figure 2(a) shows the annual articles between 2004 and 2023. Before 2017, the number of articles increased relatively slowly. However, the number of papers increased significantly after 2017. This shows that scholars have become more interested in this topic over time.

**Figure 3:**
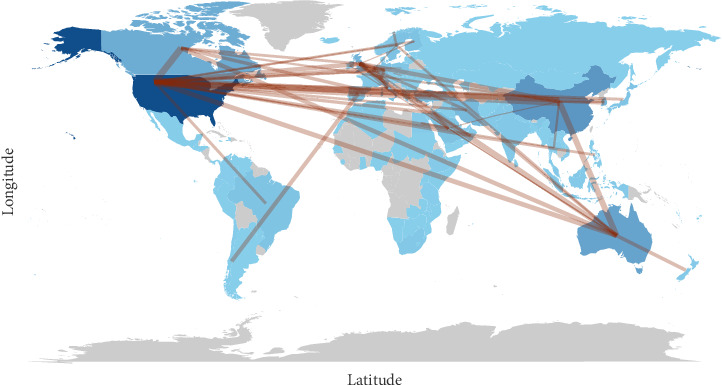
Publication distribution and countries' collaboration world map.

Between 2004 and 2023, 89 countries conducted research on nurse job satisfaction. Figure 2(b) and Table 1 show the top 10 countries with the most articles on nurse job satisfaction. The United States leads the world in the output of articles on nurse job satisfaction with 1853 articles, 43,415 total citations, and 26.51 average citations per article, followed by Australia (857 articles, 24,760 total citations, and 28.89 average citations per article) and Canada (683 articles, 30,172 total citations, and 44.18 average citations per article).  Figure 3 shows a world map of publication distribution and country collaboration, with dark blue indicating a larger number of articles and thick lines indicating more frequent collaborations between countries. According to Figure 3, the United States has the strongest connections with China, South Korea, Canada, and Australia. The United States is the country with the most developed network, with 33 countries collaborating, followed by Australia with 15 countries, and Canada with 9 countries. Although the United States has the strongest total connection strength, most of the collaborations are with developed countries, which may have ties to similar cultures, national situations, and healthcare programs.

#### 3.1.2. Articles Analysis in Journals

**Figure 4:**
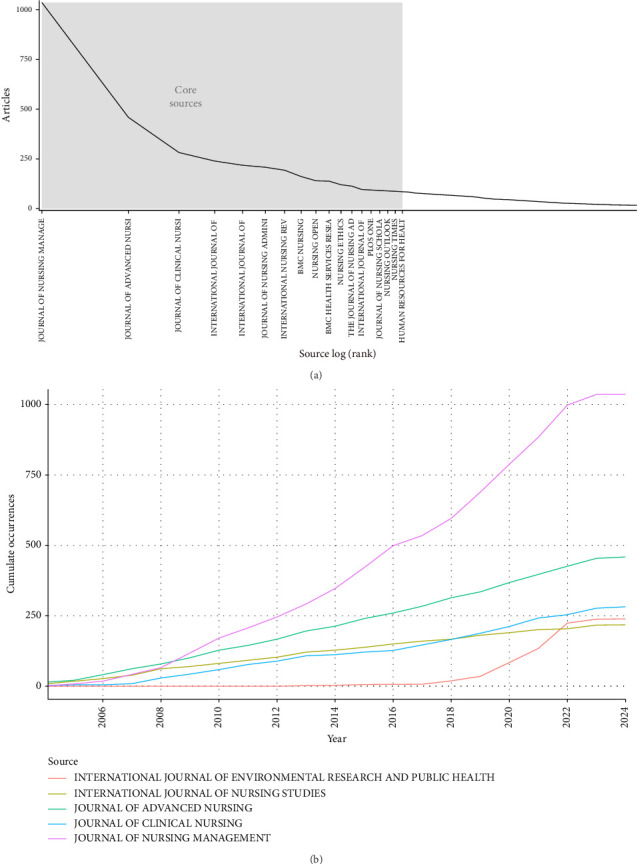
(a) Core sources by Bradford's Law. (b) The dynamics of annual cumulative publications for core journals about.

A total of 11,993 articles were published across 1735 different journals. According to Bradford's law, which posits that journals ranked by the number of publications follow a ratio of core journals to others as 1:*a*^2^ [52], these journals can be categorized into core, related, and marginal areas, with article distribution among these areas being equal. Utilizing BiblioShiny, we identified the core journals within the field of nurse job satisfaction research (Figure 4(a)). Notably, 3763 articles were published in 19 core journals, representing 31.38% of the total publications. Among them, JOURNAL OF NURSING MANAGEMENT (88 articles, accounting for 14.55% of all publications, with an average of 27.72 citations per article) was the journal with the most publications (Table 2). JOURNAL OF ADVANCED NURSING (459 articles, accounting for 3.82%, with an average of 43.19 citations per article) ranked second in terms of publication volume. Next is JOURNAL OF CLINICAL NURSING (282 articles, accounting for 2.36%, with an average of 27.45 citations per article), and INTERNATIONAL JOURNAL OF NURSING STUDIES is the journal with the most citations (218 articles, accounting for 1.82%, with an average of 91.50 citations per article) (Table 2). The H-index of the top three journals in terms of publication volume also ranks in the top three: JOURNAL OF NURSING MANAGEMENT (H-index = 35), JOURNAL OF ADVANCED NURSING (H-index = 22), and JOURNAL OF CLINICAL NURSING (H-index = 26). The United Kingdom publishes the vast majority of core journals, with the largest number of core journals published in the United Kingdom (*n* = 11), followed by the United States (*n* = 6), and Switzerland and Australia each have one core journal (Table 2).

Figure 4(b) shows the changes in the annual cumulative number of articles published in core journals on nurse job satisfaction. The number of articles on nurse job satisfaction published by JOURNAL OF NURSING MANAGEMENT increased rapidly after 2008, and in 2008, it surpassed JOURNAL OF ADVANCED NURSING to become the journal with the most articles on nurse job satisfaction. This is particularly noteworthy because JOURNAL OF NURSING MANAGEMENT has quickly become a core journal for publishing research on nurse job satisfaction since its founding in 1993. JOURNAL OF ADVANCED NURSING has also experienced a similar upward trend over the past 20 years, ranking second in terms of cumulative number of articles published.

#### 3.1.3. Articles Analysis According to Organizations

**Figure 5:**
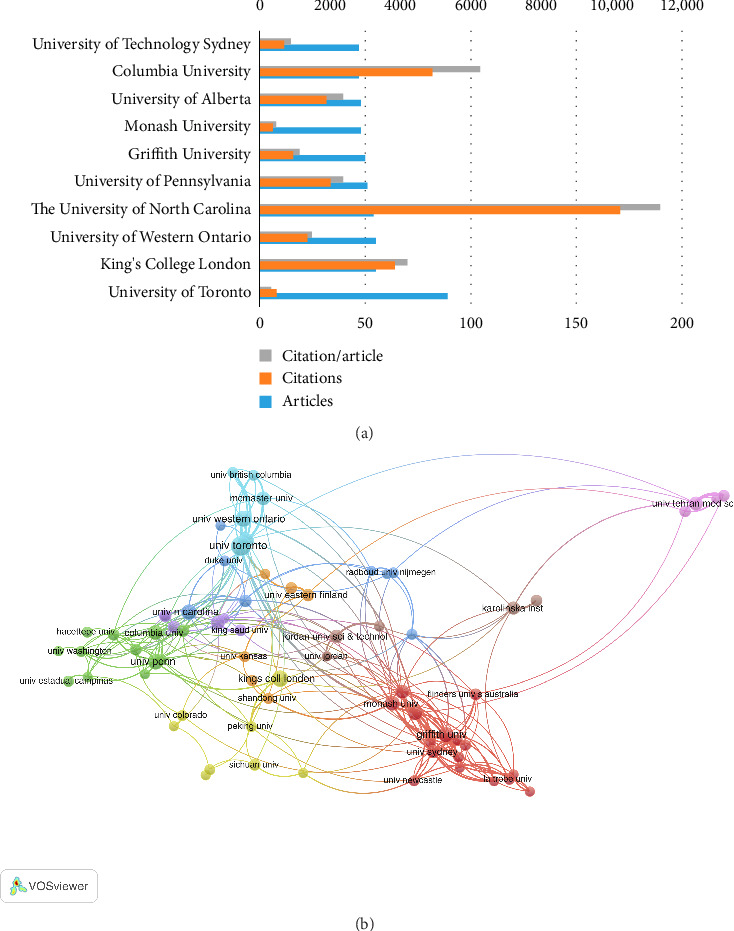
(a) The top 10 institutions of output performance in the nurses' job satisfaction field. (b) The knowledge map of the institutions' cooperation network related to nurses' job satisfaction.

In nurse job satisfaction research, 1735 institutions published a total of 11,993 articles.  Table 3 presents the top 10 institutions based on article volume. The University of Toronto leads with 89 articles (0.74% of total publications, averaging 5.45 citations per article), followed by King's College London with 55 articles (0.46%, averaging 101.22 citations) and Tel Aviv University with 8 articles (1.32%, averaging 69.91 citations). Notably, the University of North Carolina has the highest average citations per article (189.57), indicating significant impact in advancing this field. Bibliometrix also visualizes institutional collaboration; Figure 5(a) illustrates 16 institutions where node size reflects publication volume and line thickness indicates collaboration strength.  Figure 5(b) reveals eight major research clusters, showing that institutions within the same cluster tend to collaborate closely, often reflecting national proximity.

### 3.2. Research-Focused Analysis by Keywords

**Figure 6:**
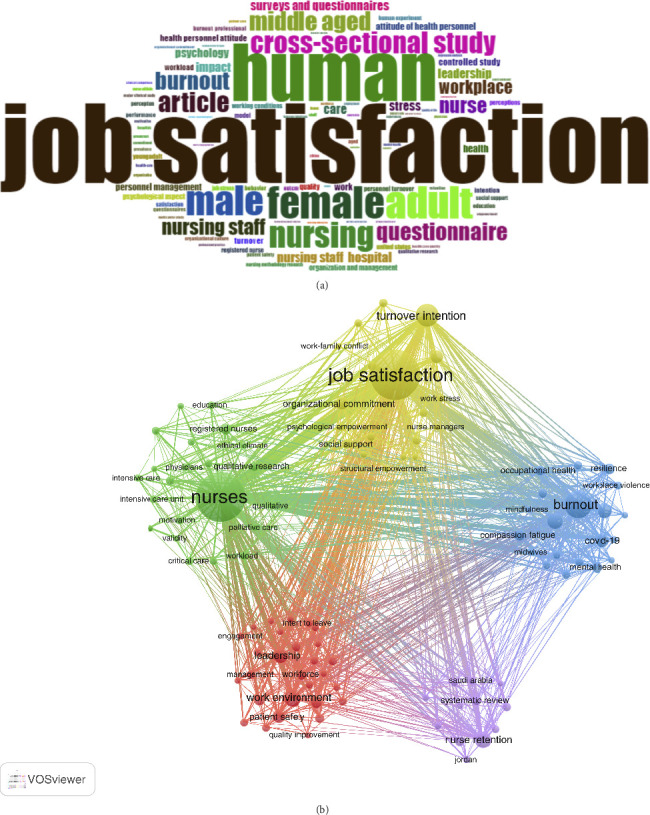
(a) Visualized word clouds of keywords. (b) Co-occurrence-keywords-network visualization.

VOSviewer indicates that 14,152 terms were used in the nurse job satisfaction study.  Figure 6(a) shows the word cloud of the top 100 terms used in Keywords Plus. The words in the word cloud become larger as their frequency increases.  Figure 6(a) shows that the most common keywords are job satisfaction (7024 times), human (5026 times), female (2863 times), adult (2829 times), and male (2608 times).

Figure 6(b) shows the co-occurrence graph of the keywords. When creating a network, the larger the nodes and words, the higher the word co-occurrence rate. Word co-occurrence is shown by the color of the nodes. According to the co-occurrence analysis, as shown in Table 4, 20 words such as job satisfaction, nurses, burnout, turnover intention, work environment, and stress are the most commonly used keywords in the research field. There are five different clusters in the co-occurrence network of nurse job satisfaction research. The first cluster (red) includes 31 items such as work environment, workforce, leadership, nurse manager, and quality of care. The second cluster (yellow) has 26 items, including job satisfaction, turnover intention, job performance, etc. The third cluster (green) includes 18 items such as nurses, workload, motivation, etc. The fourth cluster (blue) includes 12 items, such as burnout, stress, occupational stress, anxiety, mental heath, COVID-19, etc. The fifth cluster (purple) includes 7 items such as meta-analysis, systematic review, etc. The study found that clusters 1 and 3 are related to nurse management, cluster two is related to nurse job satisfaction, cluster four is related to nurse job stress, and cluster five is related to the research methods of related studies.

**Figure 7:**
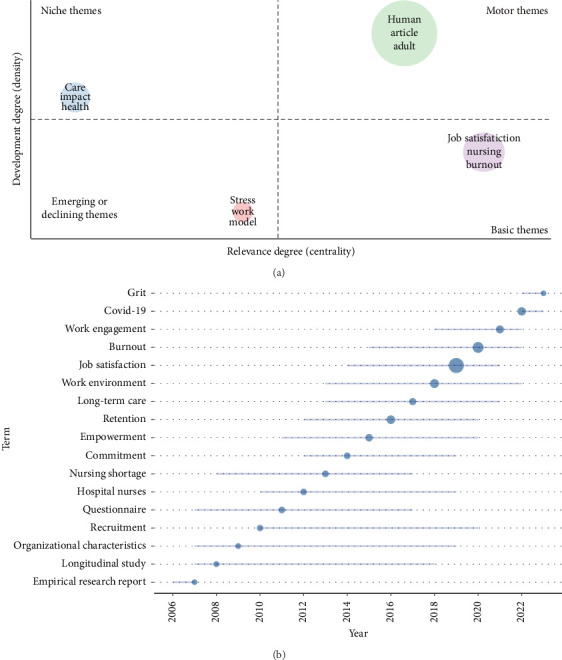
(a) Thematic map. (b) Trend topics.

Figure 7(a) shows the topic types of nurse job satisfaction research. The minimum cluster frequency is 10, the number of levels per cluster is 1, and the number of items in the thematic map analysis is 500. The sports theme in the upper right area is a very developed and important theme, consisting of three words: “human,” “article” and “adult,” which are known for their high centrality and density clusters. In contrast, niche topics in the upper left region include weak centrality and high density clusters with few but strong connections to other topics. Three terms of these niche topics are “care,” “impact,” and “health.” The basic topics in the lower right region are topics with many but weak connections to other topics, including three terms: “job satisfaction,” “burnout,” and “nursing.” Emerging or declining topics in the lower left region: clusters are topics that embody clusters with few and weak connections to other topics, including “stress,” “work,” and “model” [53, 54].

Figure 7(b) depicts the trending topics in nurse job satisfaction research over the past 2 decades. The minimum word frequency was taken as 5, while the number of words per year was taken as 1. Figure 7(b) shows that “job satisfaction,” “burnout,” “work environment,” “retention,” and “COVID-19” are the most frequently discussed topics. While “empirical research report,” “longitudinal study,” and “organizational characteristics” were the most popular topics in earlier years, other topics such as “work engagement,” “COVID-19,” and “grit” have also become increasingly popular in recent years.

### 3.3. Author Analysis

Analysis of author-related data can be used to identify major contributors and prominent scholars. In addition, co-citation and bibliographic coupling networks can be used to obtain major citation networks and research targets. Before analysis, names of the same author abbreviated in multiple ways were merged.

#### 3.3.1. Core Authors

In 1963, scholar Derek J. de Solla Price noted that 50% of publications on a given topic are typically produced by a particularly prolific group of authors, whose number approximates the square root of the total number of authors [55]. Based on Price's Law, n_max_ the number of papers with the highest productive author in a field (statistics from VOSviewer indicate n_max_ = 6) and the minimum paper of core authors in a particular subject is *m* = 0.749× $\sqrt{n_{\max}}$ ≈1.835. Consequently, in nurse job satisfaction research, authors with more than 2 articles are categorized as core authors. This group comprises 3124 core authors who have published 7567 articles, representing 63.10% of the total publications and exceeding Price's 50% standard. This suggests the formation of a relatively stable author group in this research area. Table 4 lists the 10 most productive authors in nurse job satisfaction research, while Table 5 highlights the top 10 highly productive authors in this field.

Among the highly productive authors, Spence Laschinger, Heather K. has the most articles and the most citations, with 46 articles published from 2004 to December 31, 2023, and each article has been cited 117.26 times. They focus on the research of nurse work environment, nurse burnout and nurse job satisfaction, such as the impact of workplace empowerment on job satisfaction, and the research of new nurse burnout and workplace happiness [56, 57]. Aiken, Linda H of the University of Pennsylvania is the second most cited author. He mainly studies nursing burnout [58] and the impact of job satisfaction on nursing quality [59].

#### 3.3.2. Co-Citation and Bibliographic-Coupling Network Analysis

**Figure 8:**
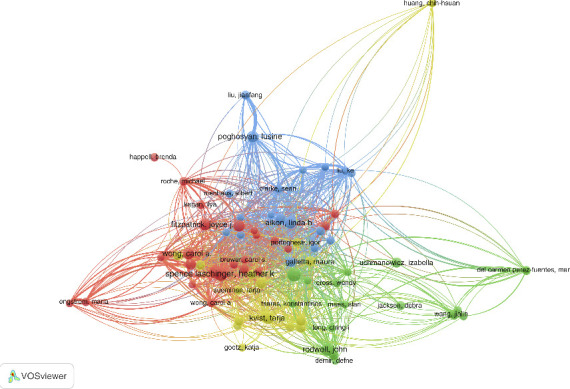
Author co-citation network analysis in the field of nurses' job satisfaction.

**Figure 9:**
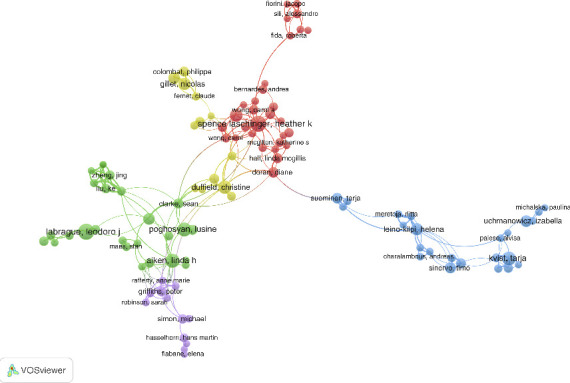
Author bibliographic-coupling network analysis in the field of nurses' job satisfaction.

The subsequent part of this study will conduct co-citation and bibliographic coupling analysis to further understand the relationship between the research focus of each scholar and their citations. Since their inception, these two important bibliometric techniques for illustrating the conceptual framework of a research field have been widely used and refined. Documents, journals, and authors can all be used as analysis units. In addition to analyzing works, author co-citation and bibliographic coupling analysis can also compare individuals with documents or journals.  Figure 8 shows that the author co-citation network is divided into four clusters based on the main research front, author quality and representativeness, and cluster size. They are: the focus of the green cluster is Aiken, Linda H, the focus of the red cluster is Labrague, Leodoro J, the focus of the blue cluster is Leino-Kilpi, Helena, and the focus of the yellow cluster is Duffield, Christine. The results obtained by the researchers from the bibliographic coupling study are shown in Figure 9, where Labrague, Leodoro J, Kvist, Duffield, Christine, and Leino-Kilpi, Helena form the network.

## 4. Discussion

This paper employs VOSviewer and RStudio for bibliometric analysis to assess the current status of nurse job satisfaction research, aiding scholars in gaining deeper insights and promoting further study in this area. Analyzing the publication trends of the 11,993 articles over the past 20 years reveals three distinct stages of evolution in the field. In the first stage (2004–2011), an average of 397 articles were published annually, reflecting a stable trend in nurse job satisfaction research. As interest grew among researchers worldwide, the number of publications increased, primarily addressing the effects of the work environment and leadership models on nurse job satisfaction [57, 60]. These early studies largely focused on the relationships between nurse job satisfaction and various work-related factors. The second stage (2012–2017) saw an increase in publication volume, with an average of 521 articles published each year, indicating a growing engagement with the topic. During this stage, research on compassion fatigue, clinical supervision, and other issues related to nursing quality and job satisfaction continued to increase [61, 62]. In the third stage (2018–2023), the research results on nurse job satisfaction showed a rapid growth trend, indicating that nurse job satisfaction has become a research hotspot, and more researchers have invested in studying this issue, especially after the COVID-19 epidemic, the work pressure of nurses during the epidemic has suddenly increased, resulting in more acute contradictions such as nurse resignation and anxiety, which has also become the main research topic during this period [63, 64]. According to the publications in the past 20 years, as time goes by, more and more contradictions are highlighted in the work of nurses around the world, and the content of research is more problem-oriented. Especially after 2018, with the arrival of the epidemic, research on nurse job satisfaction has exploded as nursing contradictions have become more acute. The content and quality of research on nurse job satisfaction are constantly improving, starting from the early stage of knowledge collection and preparation to the current rapid development stage. JOURNAL OF NURSING MANAGEMENT, JOURNAL OF ADVANCED NURSING and JOURNAL OF CLINICAL NURSING are the three journals that publish the most papers related to nurse job satisfaction. In addition to the top three journals, International Journal of Nursing Studies (although the number of articles is relatively small among the core journals, the total number of citations is high (91.50)). Scholars studying nurse job satisfaction can choose to follow these journals or submit articles to them. In addition, we found that developed countries, especially the United States and the United Kingdom, occupy an important position in the study of nurse job satisfaction. American scholar Hoppock [65] was the first person to propose the concept of job satisfaction, and the countries and organizations that published the most papers on nurse job satisfaction research are from the United States and the United Kingdom, which shows the important role played by the United States and the United Kingdom. In order to better understand the research materials, it is necessary to conduct a co-occurrence analysis of keywords. In addition, in order to identify the main authors in this field, author bibliographic coupling and co-citation networks were also used. In addition, in order to deeply understand the research content, all published articles were reviewed. The top three core authors in terms of the number of papers published are Spence Laschinger, Heather K, Labrague, Leodoro J and Rodwell, John. The research field of the main author is very consistent with the results of the co-occurrence of keywords. Hot topics help scholars gain insight into research trends and highlight important issues in a certain field [66]. According to keyword analysis, “burnout,” “staff turnover” and “nursing” are hot topics in the study of nurse job satisfaction. Evaluating the impact of other variables on nurse job satisfaction can reveal major factors such as organizational commitment style burnout and manager leadership, which reminds nursing management medical organizations to formulate policies and intervene in the main factors that raise and solve the problem of nurse turnover [67–70]. In addition, some studies have shown that nurses' job satisfaction affects the quality of nursing [71, 72]. Therefore, effective measures should be taken to improve the quality of nursing by targeting the main factors affecting nurses' job satisfaction. These views also explain why “burnout,” “staff turnover” and “nursing” have become the focus and hot spots of attention [73].

Thematic map analysis has revealed that “human,” “article” and “adult” are the motor themes in nurses' job satisfaction research. In research, the motor theme demonstrates core and developed themes together with high density and centrality [74]. It could be claimed that “human” and “adult” are vital concepts that have developed adequately in nurses' job satisfaction research. Because researchers focus on evaluating human relations value congruence and human resource management influencing nurses' job satisfaction and try to find true determinants of adult nurse practitioners' job satisfaction [75–77]. Put another way, the present emphasis of the study is “human” and further study on distinct topics is required to develop the field. Thematic map analysis's niche themes indicate that nurses' job satisfaction studies have a high density and low centrality, which include “health,” “care”, and “impact.” Comparative job satisfaction in different health care environments can discover risk impacts, such as certifying dissatisfaction with interpersonal relationships in the hospital setting and monotony as a factor in community environments [78]. Thus, future research subjects concentrating on human relations value congruence, human resource management and the impacts of adult nurses' job satisfaction in diverse healthcare settings will help the efficient development of nurses' job satisfaction research.

The trend topic analysis has illustrated which subjects have been studied most frequently over time. The topics “grit” and “COVID-19” have been popular lately. It is critical to determine the associations of grit and COVID-19 with nurses' job satisfaction, which contributes to accurately developing interventions to improve nurses' job satisfaction [79–82]. The topics “burnout,” “work engagement,” “retention,” “empowerment” and “work environment” are the most often discussed subjects. Since scholars concentrate on empowerment, work engagement, work environment and burnout as significant predictors of nurses' work satisfaction and retention [57, 83–86]. Concentrating on the topics “grit,” “COVID-19,” and “work engagement” of nurses' work satisfaction study in the future might deepen this field's understanding [87].

All of the discoveries will help to incorporate up-to-date information into the work that other researchers conduct. There has been a rise in interest in studies on nurses' job satisfaction and the number of articles has been steadily rising. Nevertheless, there remain comparatively limited studies in this field, particularly in developing nations with diverse cultural backgrounds.

## 5. Limitations

This study analyzed the literature on nurses' job satisfaction and conducted a systematic analysis of the contributing nations, journals, institutions, authors, and topics. However, it is significant to claim several limitations. First, only articles and reviews were included in the sample of literature. Consequently, the analysis was somewhat constrained. Secondly, since some bibliometric analyses use software to extract information such as article titles and keywords and then summarize and analyze them using specific algorithms, and authors from different countries may use different languages to describe the same issue, there may be inappropriate word extraction and semantic understanding, and language bias in this process. Although we have performed operations such as merging synonyms and near-synonyms during the analysis process to avoid such errors, such bias may still exist. Lastly, a few things, such as the effect of certain temporal factors and database updates, might cause a discrepancy between the real study conditions and the findings of the bibliometric study.

## 6. Conclusion

In this study, we studied articles from 2004 to 2023 so that researchers can have a comprehensive understanding of nurse job satisfaction research. We found that the research direction on nurse job satisfaction has become more established, with strong disciplinary continuity and a certain research foundation. Developed countries, especially the United Kingdom and the United States, are the main contributors to nurse job satisfaction research. First, “burnout” may be a research hotspot with development potential in the field of nurse job satisfaction. In the keyword contribution analysis, the two keywords “stress” and “turnover intention” appeared, which are closely related to the theme of “burnout.” At the same time, according to the display of the strategic coordinate map, this theme is located in the fourth quadrant of the strategic coordinate map, which means that this theme may have great development potential. Secondly, “work engagement,” “COVID-19” and “grit” are all hot topics recently. Considering the shortage of medical resources caused by the coronavirus disease 2019 pandemic, the impact of various problems in the work process of nurses on nurses' health may also be a hot topic for future research. Furthermore, international research should be given greater emphasis to investigate whether the major factors and effective interventions of nurses' job satisfaction differ between cultures and more multicenter as well as large sample studies should be conducted to efficiently improve nurses' job satisfaction.

## Figures and Tables

**Table 1:** Top 10 countries with the most articles related to nurses' job satisfaction.

Country	Articles	Citations	Average citation/article
USA	2797	74,160	26.51
Australia	857	24,760	28.89
Canada	683	30,172	44.18
China	863	17,448	20.22
South Korea	513	2389	4.66
Iran	415	5911	14.24
Turkey	383	6485	16.93
Taiwan, China	326	8682	26.63
England	529	20,032	37.87
Spain	302	6529	21.62

**Table 2:** Core sources by Bradford's law.

Journals	Article	Citations	Average citations	H-index	Country
Journal of nursing management	1036	28,714	27.72	46	UK
Journal of advanced nursing	459	19,824	43.19	25	UK
Journal of clinical nursing	282	7742	27.45	36	UK
International journal of environmental research and public health	239	4723	19.76	21	Switzerland
International journal of nursing studies	218	19,947	91.50	19	UK
Journal of nursing administration	208	7340	35.29	22	USA
International nursing review	193	4316	22.36	21	UK
Bmc nursing	161	2831	17.58	19	UK
Nursing open	140	1251	8.94	23	UK
Bmc health services research	138	3919	28.40	25	UK
Nursing ethics	120	3626	30.22	13	UK
The journal of nursing administration	113	2586	22.88	13	USA
International journal of nursing practice	96	2341	24.39	14	Australia
Plos one	94	1692	18.00	15	USA
Journal of nursing scholarship	90	5033	55.92	21	USA
Nursing outlook	88	2843	32.31	22	USA
Nursing times	88	2148	24.41	11	UK
Human resources for health	87	2773	31.87	12	UK
Nursing management	83	209	2.52	14	USA

**Table 3:** Top 10 organization with the most articles publication.

Institutions	Articles	Citations	Citation/article	Country
University of Toronto	89	485	5.45	Canada
King's College London	55	3845	69.91	England
University of Western Ontario	55	1359	24.71	Canada
The University of North Carolina	54	10,237	189.57	USA
University of Pennsylvania	51	2020	39.61	USA
Griffith University	50	951	19.02	Australia
Monash University	48	379	7.90	Australia
University of Alberta	48	1898	39.54	Canada
Columbia University	47	4908	104.43	USA
University of Technology Sydney	47	696	14.81	Australia

**Table 4:** Top 20 institutions with the most articles related to nurses' job satisfaction.

Keyword	Occurrences	Total link strength
Job satisfaction	3448	5936
Nurses	3372	6035
Burnout	1041	2379
Turnover intention	731	1629
Work environment	423	964
Stress	353	829
Nurse retention	350	718
Occupational stress	314	656
Leadership	301	611
COVID-19	266	615
Work engagement	226	482
Patient safety	222	375
Organizational commitment	210	483
Quality of care	182	389
Compassion fatigue	164	404
Mental health	164	369
Workforce	159	327
Registered nurses	153	257
Hospital	148	301
Empowerment	147	288

**Table 5:** Top 10 authors with the most articles publication.

Author	Articles	Citations	Average citation/article	Affiliation
Spence Laschinger, Heather K.	46	5394	117.26	University of Western Ontario
Labrague, Leodoro J.	45	2399	53.31	Sultan Qaboos University
Rodwell, John	37	852	23.03	Swinburne University of Technology
Aiken, Linda H.	35	3527	100.77	University of Pennsylvania
Kvist, Tarja	35	720	20.57	Helsinki City Nursing School
Poghosyan, Lusine	33	945	28.64	Columbia University
Duffield, Christine	32	1831	57.22	University of Technology Sydney
Cummings, Greta	29	1580	54.48	University of Alberta
Gillet, Nicolas	27	593	21.96	University of Tours
Leino-Kilpi, Helena	26	890	34.23	University of Turku

## Data Availability

The data are available from the corresponding author on reasonable request.

## Supporting Information

The supporting information include the search strategies and results from three databases: WoSCC, Scopus, and PubMed. Table S1 presents the search results from WoSCC, Table S2 presents the results from Scopus, and Table S3 presents the results from PubMed.
