# Supplementary material for: A Bibliometric Analysis of Nurses' Job Satisfaction From 2004 to 2023
Source: J Nurs Manag. 2025 Apr 30;2025:4285361. doi: 10.1155/jonm/4285361 (PMC12058320; doi:10.1155/jonm/4285361)
Supplement: Supporting Information — Additional supporting information can be found online in the Supporting Information section. [file 4285361.f1.docx]

**supplemental file**

Table S1 Search results of Woscc database

Search time: 2024-09-03

| #search | Query | Items found |
| --- | --- | --- |
| #1 | TS = (“nurse$”) | **251,663** |
| #2 | TS = (“job satisfaction$” or “work satisfaction$”) | **40,897** |
| #3 | #1 AND #2 | **7,281** |
| #4 | TI=(“nurse$” or “nursing personnel” OR “registered nurse$” OR “nurse administrator$” OR “nurse practitioner$” OR “nurse specialist$” OR “nurse anesthetist$” OR “nurse clinician’s” OR “nurse midwives” OR “pediatric nurses” OR “community health nurse$” OR “international nurse$” OR “male nurse$” OR “public health nurse$”) AND TI=(“job satisfaction” OR “work satisfaction”） | **1,042** |
| #5 | AB=(“nurse$” or “nursing personnel” OR “registered nurse$” OR “nurse administrator$” OR “nurse practitioner$” OR “nurse specialist$” OR “nurse anesthetist$” OR “nurse clinician’s” OR “nurse midwives” OR “pediatric nurses” OR “community health nurse$” OR “international nurse$” OR “male nurse$” OR “public health nurse$”) AND AB=（“job satisfaction” OR “work satisfaction”） | **3,720** |
| #6 | #3 OR #4 OR #5 | **7,286** |
| #7 | #3 OR #4 OR #5 and 2023 or 2022 or 2021 or 2020 or 2019 or 2018 or 2017 or 2016 or 2015 or 2014 or 2013 or 2012 or 2011 or 2010 or 2009 or 2004 or 2005 or 2006 or 2007 or 2008 (Publication Years) and Article or Review Article (Document Types) | **6,077** |

Table S2 Scopus database search results

Search time: 2024-09-03

| #search | Query | Items found |
| --- | --- | --- |
| #1 | TITLE-ABS ( "nurse$" OR "nursing personnel" OR "registered nurse$" OR "nurse administrator$" OR "nurse practitioner$" OR "nurse specialist$" OR "nurse anesthetist$" OR "nurse clinician's" OR "nurse midwives" OR "pediatric nurses" OR "community health nurse$" OR "international nurse$" OR "male nurse$" OR "public health nurse$" ) | **397,948** |
| #2 | TITLE-ABS ( "job satisfaction$" OR "work satisfaction$" ) | **36,974** |
| #3 | #1 AND #2 | **5,825** |
| #4 | TITLE-ABS ( "nurse$" OR "nursing personnel" OR "registered nurse$" OR "nurse administrator$" OR "nurse practitioner$" OR "nurse specialist$" OR "nurse anesthetist$" OR "nurse clinician's" OR "nurse midwives" OR "pediatric nurses" OR "community health nurse$" OR "international nurse$" OR "male nurse$" OR "public health nurse$" ) AND TITLE-ABS ( "job satisfaction$" OR "work satisfaction$" ) AND PUBYEAR > 2003 AND PUBYEAR < 2024 AND ( LIMIT-TO ( DOCTYPE , "ar" ) OR LIMIT-TO ( DOCTYPE , "re" ) ) | **4,263** |

Table S3 Pubmed database search results

Search time: 2024-09-03

| #search | Query | Items found |
| --- | --- | --- |
| #1 | nurses[MeSH Terms] | **101,202** |
| #2 | job satisfaction[MeSH Terms] | **29,595** |
| #3 | #1 AND #2 | **4,297** |
| #4 | "nurse$"[Title/Abstract] OR "nursing personnel"[Title/Abstract] OR "registered nurse$"[Title/Abstract] OR "nurse administrator$"[Title/Abstract] OR "nurse practitioner$"[Title/Abstract] OR "nurse specialist$"[Title/Abstract] OR "nurse anesthetist$"[Title/Abstract] OR "nurse clinician's"[Title/Abstract] OR "nurse midwives"[Title/Abstract] OR "pediatric nurses"[Title/Abstract] OR "community health nurse$"[Title/Abstract] OR "international nurse$"[Title/Abstract] OR "male nurse$"[Title/Abstract] OR "public health nurse$"[Title/Abstract] | **154,171** |
| #5 | "job satisfaction$"[Title/Abstract] OR "work satisfaction$"[Title/Abstract] | **13,685** |
| #6 | #4 AND #5 | **2,350** |
| #7 | #3 OR #6 | **6,005** |
| #8 | (("nurses"[MeSH Terms] AND "job satisfaction"[MeSH Terms]) OR (("nurse"[Title/Abstract] OR "nursing personnel"[Title/Abstract] OR "registered nurse"[Title/Abstract] OR "nurse administrator"[Title/Abstract] OR "nurse practitioner"[Title/Abstract] OR "nurse specialist"[Title/Abstract] OR "nurse anesthetist"[Title/Abstract] OR "nurse clinician's"[Title/Abstract] OR "nurse midwives"[Title/Abstract] OR "pediatric nurses"[Title/Abstract] OR "community health nurse"[Title/Abstract] OR "international nurse"[Title/Abstract] OR "male nurse"[Title/Abstract] OR "public health nurse"[Title/Abstract]) AND ("job satisfaction"[Title/Abstract] OR "work satisfaction"[Title/Abstract]))) AND (2004:2023[pdat]) | **4,438** |
